# A Survey of MPSoC Management toward Self-Awareness

**DOI:** 10.3390/mi15050577

**Published:** 2024-04-26

**Authors:** Guillermo Gonzalez-Martinez, Remberto Sandoval-Arechiga, Luis Octavio Solis-Sanchez, Laura Garcia-Luciano, Salvador Ibarra-Delgado, Juan Ramon Solis-Escobedo, Jose Ricardo Gomez-Rodriguez, Viktor Ivan Rodriguez-Abdala

**Affiliations:** 1 Posgrado en Ingeniería y Tecnología Aplicada (PITec), Universidad Autónoma de Zacatecas, Av. Ramón López Velarde, 801, Col. Centro, Zacatecas 98000, Mexico; ing.memogm@uaz.edu.mx (G.G.-M.); lsolis@uaz.edu.mx (L.O.S.-S.); lgl.hardware@uaz.edu.mx (L.G.-L.); sibarra@uaz.edu.mx (S.I.-D.); juan.solis@uaz.edu.mx (J.R.S.-E.); jrgrodri@uaz.edu.mx (J.R.G.-R.); abdala@uaz.edu.mx (V.I.R.-A.); 2 Centro de Investigación, Innovación y Desarrollo en Telecomunicaciones (CIDTE), Universidad Autónoma de Zacatecas, Av. Ramón López Velarde, 801, Col. Centro, Zacatecas 98000, Mexico

**Keywords:** multi-processor system-on-chip, MPSoC management, self-awareness, self-aware cyber-physical systems-on-chip, software-defined networks-on-chip, survey

## Abstract

Managing Multi-Processor Systems-on-Chip (MPSoCs) is becoming increasingly complex as demands for advanced capabilities rise. This complexity is due to the involvement of more processing elements and resources, leading to a higher degree of heterogeneity throughout the system. Over time, management schemes have evolved from simple to autonomous systems with continuous control and monitoring of various parameters such as power distribution, thermal events, fault tolerance, and system security. Autonomous management integrates self-awareness into the system, making it aware of its environment, behavior, and objectives. Self-Aware Cyber-Physical Systems-on-Chip (SA-CPSoCs) have emerged as a concept to achieve highly autonomous management. Communication infrastructure is also vital to SoCs, and Software-Defined Networks-on-Chip (SDNoCs) can serve as a base structure for self-aware systems-on-chip. This paper presents a survey of the evolution of MPSoC management over the last two decades, categorizing research works according to their objectives and improvements. It also discusses the characteristics and properties of SA-CPSoCs and explains why SDNoCs are crucial for these systems.

## 1. Introduction

As technology advances, the need for adequate system management becomes increasingly important. This is especially true in the case of Multi-Processor Systems-on-Chip (MPSoCs), where the number of processing elements must increase to keep up with market demands. The Internet of Things [1,2], artificial intelligence, and cloud-based digital systems [3,4] are just a few examples of technologies that have driven this need. However, each application has specific requirements, making MPSoC management a complex challenge. Adding more processing and communication resources leads to energy consumption, temperature variations, and vulnerability to different failures. Some platforms, such as the Kalray MPPA-256 [5], the Adapteva Epiphany [6], and the Sunway [7], address these issues through distributed, scalable, and heterogeneous systems. While these chips have been successful in the industry, they lack the organizational management structure necessary for migration to more powerful systems, such as a self-aware system-on-chip.

Managing an MPSoC can be challenging due to the ever-increasing demand for enhanced capabilities and the dynamic nature of new and upcoming applications. Expanding the capabilities of an MPSoC involves increasing the resources, components, and metrics it has to control, either in number or complexity. Therefore, an efficient management approach is necessary to handle functionality aspects at various levels. This includes physical elements like processing elements, memories, ports, communication, monitoring infrastructures, and nonphysical factors like process tasks, utilization time, data, and bandwidth.

This situation highlights the need for an efficient administration that can manage various aspects in a coordinated manner to achieve the system’s objectives. As a result, the management of an MPSoC should contemplate the implementation of optimization engines, i.e., control and supervision techniques and/or protocols aimed to ensure an efficient performance.

Managing the communication infrastructure of an MPSoC is critical to its overall performance. One area of significant research is the interconnection of multiple processing elements through Network-on-Chip (NoC). The NoC infrastructure involves routers that connect processing components, providing excellent scalability to MPSoCs [8]. However, with the diversity of applications and heterogeneity of new systems, the communication infrastructure must efficiently handle dynamic patterns and workloads. Poor management of NoC can lead to significant problems, such as congestion, thermal hot spots, deficient performance, and missing deadlines, so network management is essential. It should control resources such as routers, interfaces, buffers, links, packets, transmission rates, and waiting times. The system’s active supervision requires efficient implementation and control of optimization engines at various network layers.

When analyzing global system administration, it is also important to consider network administration due to their mutual interconnectedness; a network process is not independent of an application process. However, the architecture and abstraction capacity of MPSoCs allow for separate analyses of different management types while still incorporating dynamic adaptability, intelligence, and proactivity. Ignoring communication infrastructure in global management can lead to poor performance and high energy consumption [9]. Intelligent management involves monitoring and configuring control functionalities through various services [2,9]. As such, researchers have studied techniques and tools to achieve flexibility, reconfigurability, and adaptability at runtime.

Several management schemes proposed involve novel concepts like cognitive networks, self-aware systems, and Software-Defined Network-on-Chip (SDNoC) systems [2,10,11,12,13,14]. Each scheme has different structures, approaches, scopes, and optimization objectives. However, there is a research gap in this context, as no generalized modular framework is available. To address this gap, a software-based management framework is required to offer services for reuse and facilitate the development of robust embedded systems.

In this paper, we surveyed the literature on the management of MPSoC and its potential for future development. Over the past two decades, we have compiled research that specifically focuses on the management of MPSoCs. The proposed schemes have been classified based on their architecture, approach, objectives, and improvements. Our taxonomy highlights the most researched management areas and identifies those that require more attention to help overcome challenges posed by new technologies. Furthermore, we discuss the concepts of self-awareness and cyber-physical systems and their relevance to MPSoCs. Lastly, we emphasize the importance of network management and its impact on overall system management. We also suggest that the concept of SDNoC could potentially be advantageous in meeting the demanding requirements of new and future MPSoCs.

**Figure 1:**
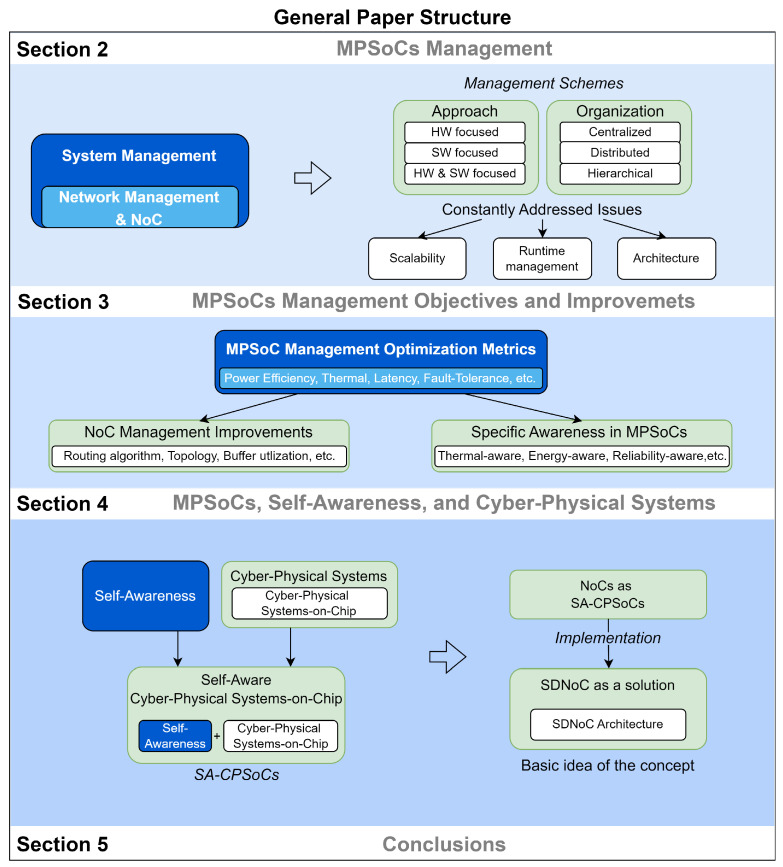
General paper structure.

The rest of the paper is organized as follows: In Section 2, we provide an overview of MPSoC management, including its concept, characteristics, and various management approaches and organizations described in the literature. We also propose a classification based on important issues that have influenced the development of MPSoCs. Section 3 classifies and analyzes different research works on proposed management schemes and their optimization objectives. We classify NoC-related works according to their main optimization metric and the most common improvement areas of NoC management. We also classify works with specific awareness, or that implemented self-x properties (focusing on adding different characteristics to the system to manage and perform processes without third-party intervention). Section 4 discusses the evolution and development of self-awareness and cyber-physical systems and their relationship, integration, and challenges in MPSoCs. The end of this section explains how a structured SDNoC architecture can help develop Self-Aware Cyber-Physical Systems-on-Chip (SA-CPSoC) through network-based system management. Finally, in Section 5, we conclude our work. Figure 1 shows the general paper structure from Section 2.

## 2. MPSoC Management

When designing an MPSoC, it is essential to manage all the interconnected processing elements within the system. With hundreds or thousands of elements, network management becomes a critical issue. While some platforms on the market offer solutions, they still require an organizational management structure capable of hosting features to monitor and control parameters within a system, aware of its state, environmental interactions, behavior, and goals. These parameters include, for example, power distribution, thermal events, fault events, security attacks, link bandwidth, routing, or traffic distribution. The following subsections present different management approaches, organizations, and issues addressed in the MPSoC research.

### 2.1. System Management

Efficient system management of an MPSoC is crucial for its overall functionality and performance. It involves optimizing processes required by applications, utilizing both hardware and software resources available in the system. These resources are complex and varied, with different levels of abstraction, including processing elements, specific process tasks, communication infrastructure, and others. Properly managing these resources involves controlling various actions like task mapping, scheduling, migration, element mapping, data distribution, and memory access [15,16].

To improve MPSoC performance, system management implements techniques and optimization engines, ranging from simple actions like turning an element off and on to complex algorithms and properties that enable self-awareness. Researchers in this area focus on specific problems like power and temperature management, QoS management, or network management to improve system management schemes [17,18].

#### Network Management

Managing the network within an MPSoC environment is crucial. Network management involves gathering information from the communication infrastructure, analyzing it, and taking corrective or preventive measures. It is a complex task to manage a network of hundreds or thousands of processing elements, and it becomes even more challenging when there is a need for runtime adaptability to handle a modern system’s workload variability. The NoC paradigm helps differentiate between computational and communication problems. However, proper network management is essential to prevent the NoC from becoming the bottleneck of system performance [2]. Hence, network management requires new control strategies that enable multiple processing elements to interact appropriately, access system resources, manage processes that require shared resources, and adapt to the environment’s variability at runtime.

The network management schemes depend on the type of communication infrastructure used, such as point-to-point links (P2P), interconnection buses, interconnection crossbar switches, or NoCs to interconnect the processing elements of an MPSoC. NoC is one of the most widely accepted MPSoC interconnection architectures. It uses traditional router and packet switch network concepts at the intrachip level. NoC architecture outperforms its counterparts in many aspects, especially regarding flexibility, scalability, and energy efficiency [8].

### 2.2. Management Approaches

Management can implement different strategies using hardware, software, or both, depending on objectives, optimization protocols, and processes.

#### 2.2.1. Hardware-Focused

Hardware-focused management schemes aim to introduce hardware elements with minimal or no use of software. These elements may include specialized monitoring agents or other dynamic management components. Hardware-focused systems are typically faster than software-based systems, as they can perform multiple tasks in parallel [19]. This approach automates management processes such as path switching, where processing speed is more favorable than the overhead that software-based implementations may introduce. However, implementing hardware-focused schemes can also lead to critical problems, such as increased area consumption, incompatibility caused by the addition of control lines, and the need for redesigning that may require longer development times [10]. Thus, the designer’s community aims to minimize hardware overhead by focusing its research efforts on developing effective hardware elements with minimal area consumption.

#### 2.2.2. Software-Focused

The implementation of software-based management systems is designed to optimize processes using software routines. This can be achieved by adding pure software agents or making adjustments at the operating system (OS) level. Although this approach adds communication and computation overhead, there are certain management processes where these overheads are unavoidable, such as congestion and flow control, which require software routines and the exchange of control messages [10]. Additionally, using silicon logic gates is generally cheaper than wires, and the development of software implementations usually involves less effort and time.

#### 2.2.3. Hardware and Software Focused

A management scheme can have a specific focus on either hardware or software, each with its own set of advantages and disadvantages. Hardware-focused strategies tend to be faster, but the ever-changing technological systems requirements are also faster than the time it takes to develop hardware. On the other hand, software-focused schemes tend to be slower, but they have the advantage of being faster to implement. Due to the challenges posed by new MPSoCs, many research papers have implemented management schemes that combine both approaches to leverage the benefits of each system. These papers establish management protocols where the congested parts performing automated tasks use a hardware approach, while the software-based approach is used for parts that require constant changes. By building the management scheme protocols offline and reconfiguring them at runtime, software allows for systems with dynamic requirements to be optimized [20]. This is especially important given the dynamism, flexibility, and harsh requirements of new MPSoCs, which drive changes in embedded systems. Therefore, studies have combined software- and hardware-focused implementations to achieve different optimization objectives with low overheads in new MPSoCs [21,22].

### 2.3. Management Organization

System management is carried out differently depending on the control assignment of the management entity/entities. How management is organized significantly impacts important characteristics such as scalability and ease of implementation. Centralized, distributed, or hierarchical management schemes are commonly used in this context.

#### 2.3.1. Centralized

In centralized management, a central entity is responsible for overseeing the entire managed system. It executes various control functions and optimization engines from a central location. Generally speaking, centralized management offers several advantages, such as deadlock avoidance due to the network overview, greater fairness in resource utilization between elements, greater simplicity of data forwarding entities, reduction in network overhead, and ease of obtaining performance metrics [23]. However, its most crucial disadvantage is the scalability problem, and its use is limited to small MPSoCs [24]. Centralized management can also reduce the system’s long-term reliability since the constant demands of attention to different actions, such as mapping or event monitoring, make it susceptible to failures [24,25].

#### 2.3.2. Distributed

Distributed management aims to overcome centralized management’s bottleneck and scalability problems [14]. To achieve this, the managed system is spatially or logically partitioned, i.e., the MPSoC can be divided into different regions (clusters), each with its management entity, or there can be one management entity per application. This strategy helps improve the system’s reliability and QoS by lightening the burden on manager entities. However, distributed managers also bring drawbacks, such as access to input and output devices that remain centralized entities, the control and allocation of cluster sizes, or the number of applications running on an MPSoC [24].

#### 2.3.3. Hierarchical

In the management field, the architecture can be centralized or distributed and may include a hierarchical organization. This organization categorizes the elements of the architecture into different operational levels and defines a hierarchy for each level. Elements at each level only communicate with those above or below their class. This hierarchical structure provides autonomy to various entities, thus enhancing their independence characteristics within the system. Hierarchical management schemes are designed to help manage ultra-large-scale MPSoCs [18,26].

### 2.4. Constantly Addressed Issues

In the design and development of MPSoCs, some aspects of their evolution must be considered. These include the constantly growing scalability issues, the runtime adaptability required by the new systems, and the paradigm changes in architectures that this demands. This approach opens the door to new challenges, such as adding self-adaptation and intelligence to future MPSoCs.

#### 2.4.1. Scalability

A key feature that current and future MPSoCs must offer is high scalability. With the increasing demand for higher performance and other new application requirements, the trend for embedded systems is to add more processing elements. However, providing high scalability can become a significant challenge for MPSoCs when talking about hundreds or thousands of processing elements. Therefore, it is necessary to consider adequate management of the MPSoC resources to ensure scalability [15], together with a layered architecture that isolates different problems to be solved independently [2].

High scalability comes with other requirements, such as power, temperature, and reliability, which become even more significant challenges for designers. In addition, incorporating intelligence in conjunction with online adaptation demands architectural improvements in new and future MPSoCs. Thus, when talking about a system with self-adaptability, scalability significantly impacts operational efficiency and can make the system objectives more straightforward to achieve [27]. Several investigations aim to increase or ensure scalability in MPSoCs. Network management is a highly investigated topic because it can become a system performance bottleneck. The recent paradigm of SDNoCs showed good scalability and network resource management. These characteristics of SDNoCs are due to their flexibility, reliability, and dynamic adaptability [2,4,9].

#### 2.4.2. Runtime Management

Today’s systems need to be flexible and adaptable to the constant changes that the dynamic behavior of new applications demands. In addition, they must provide the highest possible efficiency by taking care of the performance metrics that the application requirements dictate. Several investigations have developed schemes that allow on-the-fly dynamic management, whose objective is to provide optimization engines capable of online adapting to dynamic changes in the environment, such as varying workloads. As a result, this type of runtime-managed system has become one of the most important and crowded research areas [20]. One of the challenges for new systems is appropriately managing the available resources to perform proactive optimization, such as monitoring infrastructures, triggering events, decision making, learning algorithms, etc. Systems must perform these actions while making the appropriate trade-offs to meet the various requirements. All these actions involve the supervision of different adjustable parameters that modify the system behavior, so they must be performed at runtime to achieve optimizations according to the environment [28].

When the adaptability in MPSoC began to be studied, most research contemplated that events coming from external entities, such as the application layer or even a human operator, triggered the adaptation actions. However, current and future MPSoCs require the system to identify these events and initiate the adaptation processes, leading to self-adaptation [29].

#### 2.4.3. Architecture

While some research papers rely on traditional architectures in which they implement their proposed management of various resources, others have presented new ideas at the architectural level to improve the overall or point performance of an MPSoC. Within the diversity of research papers, some focus on making modifications at the hardware level only, and others at the software level only, but most concentrate on implementing innovations that involve hardware and software. Likewise, the new dynamic requirements and the high scalability of emerging MPSoCs demand architectural improvements at different levels. One of the most significant is related to the NoC intercommunication infrastructure. Thus, the system’s architecture must contemplate new management, control, and supervision schemes to meet the new expectations [30].

### 2.5. Evolution of MPSoC Management

MPSoC management aims to create highly dynamic environments where the constant variation of application processes demands versatile handling of hardware resources and task coordination. As the number of processing elements incorporated within an MPSoC increases, there is a need for resource management and supervision with more outstanding capabilities [30] to handle the higher power and temperature density [27], as an example. These new paradigms challenge MPSoC management, requiring different goals regarding management subdivisions such as energy, power, temperature, system reliability, QoS, security, network, etc.

Recent research into Self-Aware Cyber-Physical Systems-on-Chip (SA-CPSoCs) has demonstrated that they can solve the challenges of new and future MPSoC developments. The SA-CPSoC paradigm incorporates critical features such as self-aware, self-adaptive, learning, and reasoning capabilities within an infrastructure that enables excellent monitoring and actuation capabilities over the physical and virtual environment.

**Figure 2:**
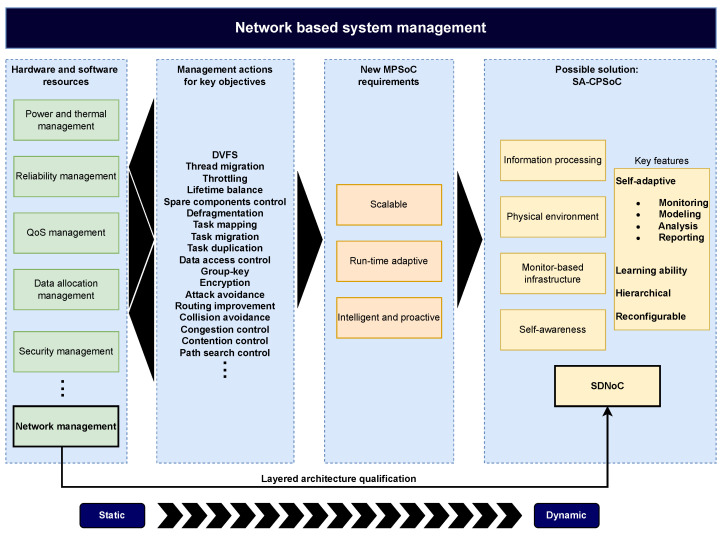
New fundamental requirements for a Self-Aware Cyber-Physical System-on-Chip through network-based system management.

Network resources management is a fundamental part of any system, and it can be a determining factor for the optimal management of the entire system. In this context, the hierarchical layered architecture paradigm of Software-Defined Networks-on-Chip (SDNoCs) can be a component that helps in developing and evolving MPSoCs towards SA-CPSoCs through network-based system management. Figure 2 shows this evolution based on the new fundamental requirements of MPSoCs and the critical features of the possible solution represented by SA-CPSoCs.

### 2.6. Summary

Modern MPSoCs present a range of new challenges for designers striving to maximize their capabilities. One of the key requirements for these systems is to add runtime adaptability while being self-aware of their state, environment, behavior, and goals. To meet these challenges, it is essential to have a management and control layer dispersed across several abstraction levels that can act according to the system’s needs, leveraging the most suitable management characteristics regarding approach, organization, and implementation status. Table 1 provides a classification of some of the most relevant research of the past two decades that focused on management issues related to MPSoCs and addressed the constantly evolving problems in this field. Although achieving greater scalability has always been one of the objectives of MPSoCs, a physical limit has been reached. Thus, it is necessary to use the available resources more efficiently and optimally based on the system’s requirements at any given time. Based on Table 1, about 65% of the research focused on adding runtime capabilities to enable the system to handle constraints and dynamism. While most of these research papers still preferred centralized schemes, there has been an increase in developments with hierarchically distributed management schemes since 2010.

These challenges have led to the design and development of new proposals for architectural improvements. Therefore, overall system management that actively involves monitoring and control strategies of the communication infrastructure may be the right path towards highly scalable MPSoCs with self-aware and self-adaptive capabilities.

## 3. MPSoCs Management Objectives and Improvements

Over the last twenty years, research papers have been primarily focused on developing and implementing techniques and procedures to improve specific optimization metrics. However, the MPSoC management is evolving towards making the system capable of simultaneously fulfilling multiple optimization objectives, paying special attention to network processes. In this context, some researchers have been working on developing “awareness” by adding monitoring and actuation capabilities to solve specific issues within the MPSoC environment. While these works were not a complete conception of “self-awareness” within MPSoCs, they serve as a crucial motivational precedent. These papers showcased systems with specific awareness to assist certain processes and improve optimization metrics. In this section, we present an analysis and classification of the optimization metrics that have been most worked on to improve the MPSoCs management. We discuss the concept of an NoC and its management and its important role in the performance of an MPSoC. We show a classification of the improvements made to the NoC environment to address the different optimization metrics of MPSoCs. Finally, we also present a classification of different specific awareness that have been worked on in MPSoCs.

### 3.1. MPSoCs Management Optimizaton Metrics

Several optimization metrics need to be considered to evaluate the performance of an MPSoC. In this paper, we considered the optimization metrics that have become more popular in the last twenty years, according to the literature. The most common metrics we focused on were power efficiency, temperature, fault tolerance, latency, throughput, security, QoS, execution time, and area.

- Power efficiency: One of the most relevant and researched aspects in the last decades is the energy consumption of embedded systems. The technological demands of new platforms have led to the integration of multiple processing elements within the same chip since they provide a level of parallelism that allows solving of the performance requirements of increasingly complex applications [145]. An NoC typically interconnects an MPSoC, which consumes a significant portion of the system power, so power consumption has become a crucial performance metric when designing [2]. An increase in specific parameters is required to meet more strict performance requirements, for example, higher operating frequencies. These demanding conditions and the workload variability of new systems increment power consumption and heat dissipation. Therefore, efficient management of these aspects has become vital in modern designs, especially in battery-operated mobile systems.
- Thermal: The on-chip temperature control of modern MPSoCs has become crucial because of its short- and long-term implications. These implications are related to high-temperature variations, which could severely affect the system’s reliability and performance [146]. These thermal conditions are especially detrimental to more temperature-sensitive systems such as optical NoCs [147]. Since conventional on-chip cooling is unavailable due to cost and space constraints, researchers are developing techniques to manage the temperature of SoCs. These management schemes also help to increase the tolerance to permanent failures, extending the lifespan of the components since the temperature is one of the leading agents that accelerates the aging effects of the SoCs [86]. Furthermore, these management techniques must be robust enough to deal with the space and time temperature distribution that the complexity of the new system NoC imposes [148].
- Fault tolerance: An MPSoC is subject to different failures affecting processing and communication links. System reliability is affected by the faults that the system may incur, so many researchers have designed architectures and management schemes to anticipate and avoid certain types of failures. The types of failures identified within systems-on-chip, especially in new MPSoCs, fall into three main categories: transient, permanent, and intermittent faults [149]. These failures are caused by effects such as soft (cosmic) errors, crosstalk, electromagnetic interference (EMI), intersymbol interference, noise, electromigration, and aging of materials [150,151]. Transient faults have a random behavior occurring in one or several execution cycles, while permanent are due to wholly damaged components that cause logic faults or operation delays. Intermittent faults have repetitive behavior and occur in the same place [149]. Several MPSoCs include spare structures to tolerate some of these failures, leveraging the increased number of processing elements. However, the increased number of processing elements sets new challenges, which makes combining management schemes with runtime system monitoring and actuation necessary to add fault tolerance.
- Latency: Communication latency within networks is defined as the time it takes for a packet to go through the network from the source node to the destination node, measured in clock cycles [2]. Latency can also denote the time it takes for some process to be performed from start to finish. For example, path-finding latency refers to the time it takes for the system to define communication paths in a circuit-switched scheme [14].
- Throughput: In a communication network, throughput is the packet rate delivered by the network, measured in bits per clock cycle. This metric is based on the count of packets reaching their destination within a given time interval for each source–destination link pair. Throughput is also defined as the maximum load the physical network can handle. Current MPSoCs demand higher requirements for applications running task parallelism with intensive information exchanges [86]. Thus, the system must offer throughput guarantees to meet the deadlines incurred by demanding applications [115]. Resource management focused on controlling certain variables, such as congestion or network traffic, can significantly benefit this performance metric.
- Security: Security has taken an important role in recent years within the MPSoC environment. New paradigms, such as IoT, seek the massive integration of devices sharing resources, making them more vulnerable to malicious attacks. Most MPSoCs are interconnected by NoCs that have access to all system resources and information, so most attacks are aimed at corrupting the NoCs through malicious software. This malicious software degrades the overall performance of the system and its services, breaches sensitive information, and can even cause failures in its components, such as routers or switches. For this reason, researchers are developing various management schemes to manage particular resources more efficiently. These schemes include the use of private keys and agreements, runtime monitoring of network traffic, and dynamic adaptability of the system to provide support against the most common attacks such as [37] denial of service attacks (DoS attacks), distributed time attacks (DTA), spoofing, tampering, repudiation, information disclosure, or privilege elevation.
- QoS: Quality of service encompasses a series of specific requirements linked to optimizing particular metrics for a given expected performance. Therefore, QoS is related to providing certain guarantees for specific requirements such as reliability, bandwidth, or latency in scenarios involving restrictions and limitations [150].
- Execution time: Many applications require performing several subprocesses simultaneously within an MPSoC interconnected by an NoC. The execution time and energy efficiency of these subprocesses are vital for real-time applications and various domains. The execution time of these subprocesses depends on the general state of the system, the critical subprocess, the available resources, and their management [40,85]. Resource management can directly influence the execution time of various applications. A way to achieve this is by employing self-awareness and monitoring-based frameworks to add adaptability to the system [66,85]. Another method can be migrating tasks to contiguous processing elements [127], or managing shared data in memory (Scratchpad-memory) [40].
- Area: The need to increase the capabilities of MPSoCs leads their components to occupy more space. However, the technological trend is to develop more powerful, smaller devices. Thus, a critical research and development objective is to keep area consumption as low as possible. Some research papers have proposed management schemes that include energy consumption, throughput, latency, and scalability thinking in area consumption.

Table 2 classifies the management research papers from the past two decades based on their optimization metrics. The table shows the metrics in order of research paper count, from the highest to the lowest, from left to right. At the end of Table 2, two additional columns are presented to highlight the research trend focus. The first identifies an NoC-based approach that recognizes papers that have addressed network-related topics, while the second identifies a self-awareness approach that recognizes papers that have mentioned some self-related properties explicitly (self-x properties).

After analyzing Table 2, we examined different time periods to identify the research trends related to the number of papers on metrics and design paradigms within the MPSoCs. Our study considered all research in recent years, narrowing the range from the last twenty years to the last five years in five-year intervals. The results are presented in Table 3.

According to our study, power and temperature are the main concerns with the highest percentage of research papers, even though works aimed at improving characteristics and solving related problems have slightly decreased over the years. The research trend for managing other metrics in MPSoCs has had its ups and downs but remains a reference for research in the field. For example, metrics such as fault tolerance have been trending almost entirely upward since they are closely related to overall system reliability and performance, or security, which has also gained importance due to the increased vulnerability of new systems to potential attacks.

On another note, NoC-focused research accounted for more than 50% of the papers analyzed, highlighting the importance of this paradigm as a communication infrastructure for MPSoCs. For this reason, various metrics appearing in our classification are closely related to NoC issues. One of these NoC-related metrics is throughput, which has declined, but it is still a major issue as NoC capacity remains a crucial issue. Latency is another NoC-related metric that has remained a research topic due to new application system requirements that demand specific deadlines for information exchange capacity within the NoC.

Finally, a research topic that has become relevant in the MPSoCs field is self-x properties. The upward research trend in self-x features, with almost 50% of research papers investigating these features in the last five years, reflects the need for systems to become self-aware. Research topics associated with this trend focus on adding different characteristics to the system to manage and perform processes without third-party intervention.

In the following subsections, we discuss the importance of NoC management in MPSoCs and present a classification of those research papers that specifically present improvements in NoC management. We also present a classification of those research papers that introduce specific awareness incorporating some self-x properties.

#### 3.1.1. NoC Management Improvements in MPSoCs

**Figure 3:**
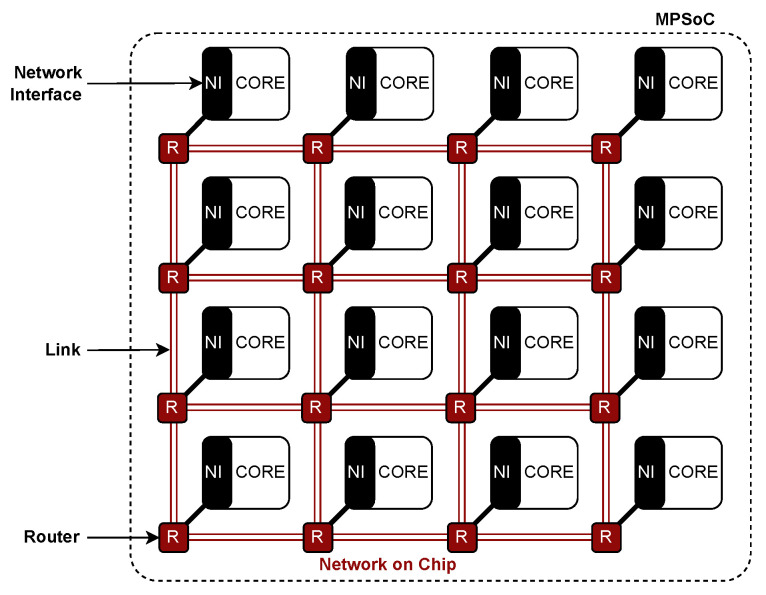
Typical Network-on-Chip architecture.

An NoC is a packet-switching network using routers to interconnect the processing elements inside an MPSoC, as shown in Figure 3. It is a conjunction of micronetworks enabling communication between the processing elements, each including a network interface. The network management implicit in an NoC is fundamental to ensuring efficient and reliable performance of the communication infrastructure of an MPSoC. NoCs architecture adds parallelism to the information flow [29], which, in conjunction with multiple processing elements, allows MPSoCs to run various types of applications [9]. This makes the control and management of resources, such as task allocation and coordination of the communication infrastructure, critical to the performance and power consumption of the system. Although the NoC paradigm allows its functionality to be widely scalable and flexible, adding simplicity and modularity to the MPSoC design by decoupling communication and computation [2,23], it is also true that it faces significant challenges with the shrinking trends of its components, especially in terms of reliability and power consumption [27].

NoCs adopt many of the concepts of traditional networks, so their management is based on a conventional network architecture consisting of three main planes: data transmission, control, and management. Basically, the control plane integrates the decision-making processes regarding the exchange of information between processing and storage elements based on established protocols, i.e., it controls the functionality of data transmission plane entities such as routers, switches, and interfaces. On the other hand, the management plane allows monitoring and configuring of the control functionality through software services [2].

NoC has become the communication infrastructure of choice for MPSoCs due to the capabilities and advantages it offers. In this context, the NoC management has been gaining importance in recent years, as shown in Table 2. NoC-related research papers are focused on improving one or more of the NoC management features like routing algorithm, network topology, buffer utilization, buffer fluidity, etc., where these improvements are aided to enhance some of the system optimization metrics. Table 4 shows a classification of the NoC-related papers according to their main optimization metric and the three most common specific NoC management improvement areas in accordance with our investigation: routing algorithm, topology, and buffer.

- Routing algorithm: In an NoC, a routing algorithm is a procedure whose main objective is to forward and distribute packets from source to destination through the best path available in the MPSoC [194,204]. The related works are commonly aimed at solving the usual routing protocol problems, such as deadlock, livelock, congestion, or network faults [204]. Some of these works implement modern techniques to deal with these problems, for example, by using adaptive routing to find the shortest path and preventing possible changes in the network [194], or in other cases, by using self-properties to find a path within a faulty network [195].
- Topology: An NoC topology represents the physical and logical distribution of the channels and nodes within the network, and, normally, its design has a cost-performance impact in the NoC [160]. The most common NoC topologies are mesh, torus, tree, polygon, and butterfly [190]. In this context, researchers have worked in developing new topologies or modifying existing ones to implement communication infrastructure improvements like circulant topology [203] and Butterfly-Fat-Tree topology [183] for improving fault-tolerance, honeycomb topology [160] for improving network-cost, WK-Recursive topology [192] for improving power efficiency and latency, RicoBit topology [190] for improving latency, or Spidergon topology [205] for improving structure and modularity. Also, new development includes not only 2D topologies but also 3D topologies [142,143,161,173,174,189,190].
- Buffer: NoCs use buffers to store transmitted packets for a short period of time within a router before they are processed to be forwarded. Some works have focused on improving certain aspects related to buffering, such as prioritizing flits forwarding through buffer fluidity levels awareness [10] or reducing underutilized buffers through new buffer design and switches’ operation monitoring [198].

#### 3.1.2. Specific Awareness in MPSoCs

The awareness integration within the MPSoCs field is one of the most recent challenges, so many researchers have implemented specific awareness to help improve the performance of these systems. In a general definition, self-awareness alludes to an entity that is capable of being aware of its state, condition, situation, and environment [144,206]. In this context, we refer to specific awareness to the partial application of the term self-awareness in MPSoCs, i.e., that the system only knows very specific things. Although the research focused on specific awareness is far from the ideal conceptualization of whole-system self-awareness, these works have conformed a necessary precedent to identify the path toward self-aware systems. Table 5 presents the classification of the research papers implementing specific awareness. The table shows, from left to right, the type of specific awareness with the highest number of research papers to the one with the lowest number. Ultimately, we also present an extra column that identifies papers focusing on NoCs.

The purpose of Table 5 is to show the number of papers dedicated to investigating awareness within the MPSoCs. Likewise, this table helps us identify the specific types of awareness studied and their intended purpose. Table 5 is closely related to Table 3 since we can observe that the most significant number of papers have been directed to the system to focus awareness on aspects such as temperature and energy. Researchers focused about 50% of these papers on NoCs-related issues. In the NoCs context, much of this specific awareness involves managing network resources, such as traffic-aware, network-congestion-aware, network-contention-aware, workload-aware, buffer-fluidity-aware, and loss-aware (optical networks) systems. We also found papers focused on adding other types of awareness related to different aspects of the system, such as reliability, the kind of application executed, environmental fluctuations, and QoS.

- Thermal-aware: Thermal-aware research is concerned with implementing techniques focused on the system not exceeding the set temperature limits while dealing with its constraints and varying processes and workloads. In addition, they involve addressing challenges immersed in temperature behavior management techniques that are related, for example, with limitations on the number of sensors that can be included in the system or with the performance impact of continuous monitoring of the temperature distribution across the chip [148].
- Energy-aware: Since one of the main goals of modern systems is to maximize battery lifetime, researchers have aimed to improve the power performance of MPSoCs. One problem is predicting the application’s behavior for adequate energy management, either by implementing known techniques or by generating new and improved ones. Consequently, some research papers have included a methodology in which the system monitors and acts on energy consumption, allowing it to improve several aspects. For example, through learning policies, the system can better respond to dynamic changes in applications [186] and to NoC processes that impact energy consumption the most [52]. Another way is by monitoring the strategies of other techniques, such as task replication, which, while improving system reliability, can also increase energy consumption too much [184].
- Reliability-aware: Within the MPSoC environment, reliability is related with the system’s ability to respond to possible failures, so the more prepared it is to resolve failures, the more reliable it becomes. Although MPSoCs are exposed to different types of faults (see Section 3.1—Fault-tolerance), research has identified three main types that affect the reliability of electronics: manufacturing defects, constant random failures, and failures due to aging of materials [197]. As a result, monitoring the system’s reliability is necessary, which consists of adequately managing the MPSoC resources, i.e., keeping the system aware of the communication infrastructure, application processes (allocation and execution of tasks), and memory performance. In this way, a reliability-aware system constantly acts at different levels to ensure specific QoS requirements.
- Traffic-aware: Traffic-aware research focuses on monitoring the amount of information exchanged through the communication infrastructure, usually an NoC (communication through routers). This runtime monitoring can be focused on specific key regions or distributed across the NoC. Traffic awareness allows the innovation and implementation of techniques applied in different communication processes, such as arbitration mechanisms that improve network latency [95] or routing algorithms that increase throughput [189].
- Congestion-aware: The congestion of the communication infrastructure of an MPSoC depends on several factors, which, in the case of NoCs, is closely related to the amount and type of traffic, latency, and network throughput. In addition, the characteristics and properties of routing and arbitration schemes play an important role in network congestion. Therefore, monitoring various metrics can improve network performance, such as leveraging information from buffers, which allows dealing with dynamic traffic loads through cognitive processes and control techniques [10]. Another improvement is identifying data flows that congest the network in certain areas or situations and subsequently avoiding them, resulting in considerable energy savings [82].
- Environment-aware: Environment-aware research explores the interaction between hardware and software components at different system levels and then implements management improvements with diverse objectives [68,133].
- Application-aware: Most NoC designs within MPSoCs do not consider the types of applications and their requirements [168]. This situation can degrade the performance of the entire system. Therefore, some papers have proposed strategies that involve application awareness at the network level, for example, by identifying the optimization metrics to which they are most sensitive and then classifying and treating them accordingly [164]. Another solution is monitoring their communication patterns and balancing the traffic load between resources by estimating routing demands [168]. In other cases, implementing continuous learning of application profiles allows the system to apply preventive and corrective actions to aid with QoS management [29].
- Workload-aware: The tasks of the application(s), running at any given time, define an MPSoC’s workload, making it a highly variable parameter. Generally, the NoC of the MPSoC reflects the implications resulting from workload variability, since if the NoC is unaware of these variations, it may fail to manage its resources. Therefore, workload awareness is highly beneficial and can be applied to improve network performance. For example, it can enhance routing algorithms by evenly distributing NoC traffic among active resources [179]. It can also help self-recover systems from failures by identifying free processing elements at a particular time [30] or the unpredictability of runtime workload by aiding dynamic memory management [81].
- Contention-aware: Contention-aware research involves the system being aware of the competition in the NoC to perform intercommunication between processing elements. Given the large number of processing elements in MPSoCs, there are more concurrent parallel intercommunications, so if there is no contention-free access scheme, contentions can degrade NoC performance. Consequently, considering network contentions can help achieve different optimization objectives. This type of specific awareness can be achieved through task mapping and scheduling in communication channels [106,163], and, likewise, in optical NoCs leveraging the flexibility of adaptive routing schemes [193].
- QoS-aware: QoS-aware research aims to provide information that helps appropriately manage available resources to meet the application requirements. This type of specific awareness can be implemented, for example, to achieve coordinated management involving the QoS of multiple resources within a class-of-service-based architecture [138]. Similarly, QoS monitoring allows for self-adaptive QoS management at runtime, providing better resource understanding and a reactive and proactive decision-making capability [29].
- Loss-aware: In optical NoCs, light signals usually suffer losses while propagating through the waveguides. This condition usually requires higher power injection into the laser to counteract these losses and avoid transmission errors. Generally, the power setting of transmission lasers does not consider these losses, so a system adding the awareness of them can increase communication and energy efficiency through adaptive runtime power setting [185].
- Fluidity-aware: Fluidity awareness refers to understanding the fluidity in the NoCs router buffers. Researchers implement active buffer monitoring to approximate the flit fluidity levels, which helps to improve flow and congestion control [10]. A flit is the smallest entity into which information exchanged over the network is divided. In addition, fluidity awareness allows for flow prioritization, which in turn allows for better management of network resources and prediction of dynamic traffic behavior.

## 4. MPSoCs, Self-Awareness, and Cyber-Physical Systems

The fusion of MPSoC with the state-of-the-art concepts of self-awareness and cyber-physical systems represents the evolution of traditional MPSoCs towards platforms that incorporate highly autonomous and self-adaptive management [12,13]. Combining these concepts within the SoC field allows us to assimilate a system capable of managing and adapting its autonomy by learning from its runtime environment. In the following subsections, we present and describe the concepts of self-awareness and cyber-physical systems.

### 4.1. Self-Awareness

The term self-awareness is used in many fields of science and is broadly concerned with an entity being aware of its own state, condition, situation, and environment [144,206]. In 2013, as an important precedent, Kornaros et al. [17] surveyed research on intelligent systems through dynamic monitoring and management techniques. In their work, they also establish the characteristics that this type of system should have. These characteristics are proactive management and monitoring since they allow decisions at runtime based on such evaluations and make the system capable of adapting in real-time. They mention that online monitoring is the fundamental tool for a system to have adaptive runtime management. They predicted that the features of new MPSoCs had to include monitoring platforms with reconfiguration capabilities and programmability of their components.

**Figure 4:**
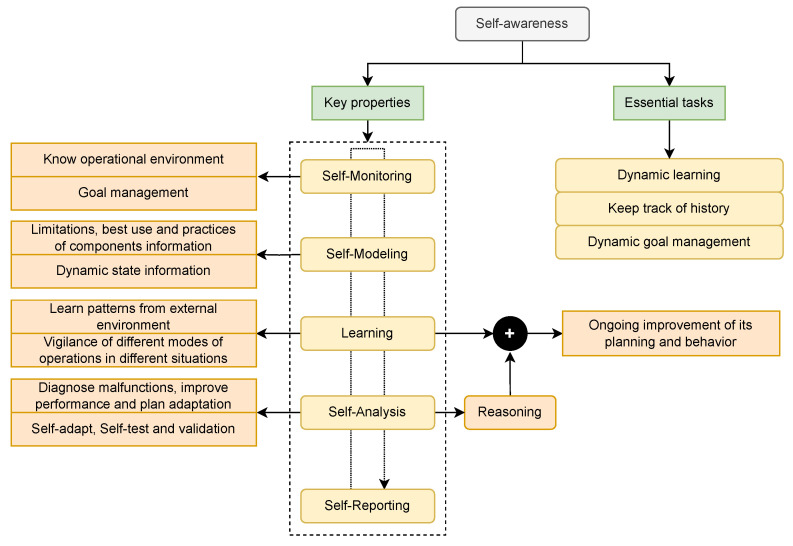
Key properties and tasks of a self-aware system based on information presented in [12,206].

In the last decade, although some researchers have tried to define and introduce the concept of self-awareness in the MPSoC field, many researchers have applied the concept partially. Thus, as Jantsch et al. [206] and Dutt et al. [209] mentioned in their work, it was necessary to lay the foundations of what it implies and understand its scope and benefits. The concept of self-awareness in computational systems involves not only proactive monitoring that provides information on the current state of the system and self-adaptability but also having an awareness of the model of the static and dynamic properties of the system, and thereby making decisions that trigger actions in the direction of the operation objectives [144,206]. Thus, a self-aware system can automatically adapt to changing environmental conditions and demands to meet its goals by constantly modifying its behavior and updating its components and resources [144]. Self-aware systems are intended to continuously perform a series of actions. They learn operation patterns based on different system situations and use reasoning to make decisions based on self-analysis at runtime. This is achieved by being aware of the hardware infrastructure and software architecture. Bellman et al. [12] defined the following terms as key properties of a self-aware system: self-monitoring, self-modeling, learning, self-analysis, and self-reporting (Figure 4). In addition, three essential tasks stand out from a self-aware system: dynamic learning, dynamic goal management, and keeping track of history [206].

A system that integrates self-awareness is a system whose behavior is based on a constant, updated, and detailed monitoring of its own state, learning and reasoning from the interaction with its environment, and acting according to the specific objectives of the system. Therefore, self-awareness is a feature that can help the system better manage and understand its behavior, which invariably improves the use of available resources, resulting in greater efficiency [206].

### 4.2. Cyber-Physical Systems

It is impossible to separate physical and computational processes in a computational system, as what happens in both affects each other. Thus, the computational and network entities continuously control and monitor the physical processes. The integration of computational and physical processes is represented by cyber-physical systems (CPS) [210]. These systems constantly interact with their physical environment. They must deal with aspects such as material degradation and aging, considering the constraints of their internal resources, such as computational and memory capacity [12].

#### Cyber-Physical Systems-on-Chip

The Cyber-Physical System-on-Chip (CPSoC) concept incorporates the cyber-physical systems paradigm into the SoCs field. While the design of a traditional MPSoC does not specify the explicit, monitored, coordinated, and controlled relationship of computation and communication operations with the physical environment, a CPSoC architecture incorporates an entity in charge of control, communication, and computation which interacts with the physical processes at runtime [79,107]. In addition, the structured architecture of a CPSoC allows the system to monitor different aspects through the different layers, providing essential information to deal with process variabilities. This information adds adaptability to the system, as it can be used in mechanisms capable of acting at various levels [144].

**Figure 5:**
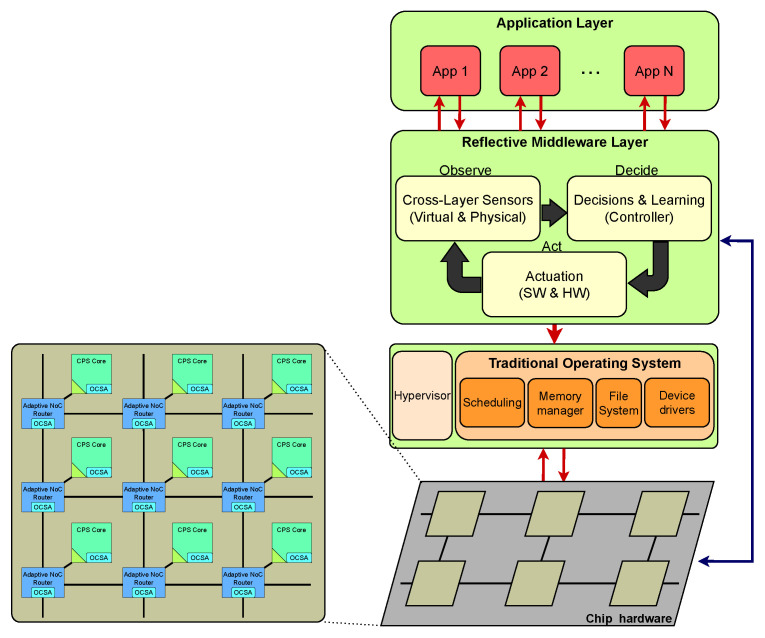
CPSoC architecture [211].

Sarma et al. [211] defined the base architecture of a CPSoC (Figure 5), where they divide it into several abstraction layers interacting with a platform composed of different sensors and actuators, whose objective is to provide the control and management of the cyber-information and the physical environment of the chip. They achieve this by using the Observe–Decide–Act (ODA) paradigm in combination with adaptive and reflective middleware that includes adaptive NoCs and some degree of self-awareness. A CPSoC platform provides a computing framework that enables the simultaneous control and management of data processing and physical environment manifestations. Thus, physical and virtual sensors and actuators ensure data reliability by considering aspects such as power, temperature, degradation, and system performance. Sarma et al. [211] mentioned that adaptability and self-awareness can be added to each abstraction layer through these physical and virtual sensors and actuators (a combination of software and hardware).

### 4.3. Self-Aware Cyber-Physical Systems-on-Chip

MPSoC design has moved toward submicron platforms, with increased complexity and design requirements. These platforms integrate many processing elements into increasingly heterogeneous systems for higher functionality and performance. New applications demand increased capabilities from MPSoCs, so computation and intercommunication between their components must be faster and more efficient [79]. They must also maintain acceptable optimization metrics such as power, temperature, and energy. Thus, new MPSoCs must be systems that constantly deal with variable processes and dynamic runtime objectives while maintaining high reliability, security, and efficiency [212]. In this way, self-aware Cyber-Physical Systems-on-Chip (SA-CPSoCs) represent a suitable solution to these demands, being CPSoCs which add self-awareness. These characteristics make an adaptive and dynamic system possible, aware of its condition, state, behavior, and what is happening in real time in its physical environment, all with little or no human intervention [12,79,144,210,213]. Thus, the design of the SA-CPSoCs allows a significant increase in adaptability through highly autonomous and intelligent system management.

**Figure 6:**
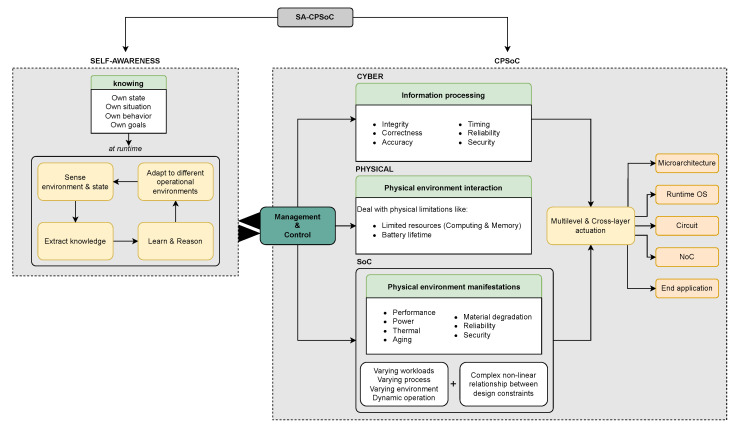
SA-CPSoC characteristics, based on information presented in [12,144,206].

The research of Bellman et al. [12] is one of the most recent works on self-aware cyber-physical systems. It defines them as self-managing systems that know their state, situation, behavior, and goals through knowledge extraction from their physical and virtual environment. These systems are supposed to learn and reason at runtime to subsequently make fast and effective adaptive decisions autonomously in the face of unexpected events. Thus, the addition of these characteristics within a system-on-chip leads to SA-CPSoCs. In this way, SA-CPSoCs increase management and control capabilities and represent the evolution of MPSoCs by adding learning and reasoning mechanisms that allow the system to self-model based on the continuous understanding of its static and dynamic properties to anticipate and correct faults. In their research, Dutt et al. [144], Jantsch et al. [206], and Bellman et al. [12] described the key properties and characteristics that CPSoCs that aim to add self-awareness must meet, considering the development and implementation challenges that this task implies (Figure 6). In addition to the self-awareness features, an SA-CPSoC monitors the behavior of different variables between the abstraction layers of the system, using these data to implement statistical prediction and learning models. These models are used by the actuation mechanisms that perform adaptations at different system levels, such as in the intrachip communication system or in the operating system. These operations must be performed with awareness of the information processing, physical manifestations, and updated system objectives. The properties and characteristics of SA-CPSoCs aim to improve the system’s autonomy, making it capable of self-managing its resources and enhancing its utilization at runtime.

#### Prospects, Future Development, and Challenges of SA-CPSoCs

Emerging MPSoCs need to deal with increasingly heterogeneous systems and hostile environments. In their conception, SA-CPSoCs prove to offer the capabilities required by modern and future applications where the system is required to have full control and fresh information of all its resources to act accordingly at runtime. The accelerated technological progress and its necessities force the development of tools and systems with greater capabilities, and the field of MPSoCs is no exception. The progress made in recent years in the agreement of the definition of self-awareness in this field has laid certain foundations for the development of such systems. As mentioned by Bellman et al. [12] in their work, the application of self-awareness in its entirety may not be the most profitable for all cases, and some applications may only require some of its characteristics. It is, therefore, necessary to think in the future about the design of SA-CPSoCs as a generalized design that can be applied to a wide range of applications, rather than thinking about adding self-awareness to an individual system [12]. In this context, these systems must provide a flexible infrastructure that allows for the adoption and organization of processes inherent to a self-awareness nature. However, there are several challenges to overcome to make SA-CPSoCs possible in fullness of their definition, especially in this resource-constrained system. Table 6 shows the challenges identified by some researchers who have worked the most in this area.

### 4.4. NoCs as Self-Aware Cyber-Physical Systems

NoCs face several design and implementation challenges in modern MPSoCs where the dynamic workloads demanded by new applications impose the necessity to place several NoCs in parallel to interconnect many entities, such as processing elements, memories, and ports, to meet system performance requirements. This workload variability involves different traffic patterns within the network, which makes NoCs unpredictable, leading to system instability if proper resource management does not exist. In addition, these factors add uncertainty at design time because it is practically impossible to know all the scenarios the system would face during its operation, which decreases the efficiency and performance of a predefined design for specific applications.

For this reason, the necessity arises to design NoCs with adaptive capabilities, allowing them to meet various important requirements such as power consumption, reliability, security, response times, and performance. These requirements must be satisfied even when the system’s conditions, like temperature and voltage, vary during the execution of different processes. A cyber-physical NoC with self-aware capabilities may be the first step toward SA-CPSoCs. In this context, an NoC can apply reconfiguration actions based on up-to-date knowledge of the system status and situation (active monitoring), actions such as dynamic bandwidth adaptation, routing algorithms, arbitration policies, topology, and so on, or the application of techniques such as throttling, DVFS, or clock gating to adjust power consumption at runtime. These actions make it possible to meet performance objectives or to achieve the best possible optimization by making the necessary trade-offs following the system capabilities and constraints and offering certain guarantees [2].

### 4.5. SDNoC as a Base Architecture in the Many-Core Era

A key component for the capable and efficient management of an MPSoC is the communication infrastructure. An MPSoC communication infrastructure aims to enable communication links between the system components, taking the information from a source to a destination entirely. This sharing of information is critical for the correct operation of the MPSoC, so the infrastructure must be sufficiently robust and guarantee the necessary communication resources for each application. If the communication infrastructure cannot meet the application resources requirements such as bandwidth, throughput, latency, traffic, waiting time, or utilization time, it can become a serious problem affecting the entire system’s performance.

In the MPSoC environment, four basic communication infrastructures are commonly found: point-to-point interconnection (P2P), shared bus interconnection, crossbar switch interconnection, and NoC. Although all these infrastructures’ general objective is to communicate the multiple processing elements within the system, each has different capabilities. P2P interconnection implies dedicated communication links between each pair of elements, i.e., there is only direct communication between two elements where a handshake protocol controls the traffic. Shared bus interconnection implies that all elements share a communication bus controlled by an arbiter, but there can only be one active link between two elements at a time. Crossbar switch interconnection implies a communication backbone controlled by an arbiter where there can be several communication links between several elements simultaneously, as long as there is not more than one link for the same receiver. Finally, NoC infrastructure implies a packet-switching network through interconnected routers throughout the MPSoC, enabling possible communication between all the elements of the system.

Compared to its counterparts, the advantages of an NoC make it the most feasible communication infrastructure for MPSoCs. These advantages lie primarily in flexibility, scalability, and energy efficiency [8]. The NoC communication infrastructure can make the design of MPSoCs faster and more efficient, allowing the implementation of distributed schemes. For example, the system can have multiple communication links transmitting information from different segments instead of concentrating the information in a shared bus. These features contemplate new allocation, control, and monitoring challenges.

The variability of the processes and workloads of modern systems requires the NoC to implement control of adjustments that adapt the communication resources in the best possible way. This control needs to be autonomous and at runtime, i.e., the system must identify those events that may trigger adaptation actions and make decisions about them at runtime [29]. An NoC must consider, for example, network traffic, congestion, contention, and fluidity when making adjustments. Likewise, the new MPSoCs require that these actions consider the unpredictability of their processes and add intelligence to allow them to anticipate and act accordingly [27].

These characteristics are part of SA-CPSoCs, and adding them to new systems is a titanic and challenging task to carry out in a system holistically, so they must be implemented in a modular fashion with a hierarchical organization. In this way, the problem is divided into smaller and less complex dilemmas that allow progress toward the final objective. Although the resources of an MPSoC can be very varied, we can divide them into computational and communication resources [29]. The former is regarding the data processing in the information processing and storage elements. The latter relates to sharing information through the network, i.e., data transmission and reception. Management of NoC resources is a critical and fundamental part of managing an MPSoC. The NoC is responsible for interconnecting hundreds, and even thousands, of processing and storage elements within an MPSoC through reliable and secure means, allowing correct and efficient operation according to the system requirements.

In the paradigm of cyber-physical systems, control, computation, and communication are closely related. Similarly, in the management plane of an MPSoC, the management of the NoC cannot be excluded. Therefore, their interaction must be taken into account during decision making in a self-adaptive system. We believe that managing an MPSoC based on network processes increases the control and management capabilities of the entire system. The distributed scheme of an NoC allows the system to divide problems throughout the network while communication and computing resource management are also distributed. In this context, advances in developing SDNoC architectures can help achieve self-adaptive cyber-physical systems.

#### 4.5.1. SDNoCs as a Solution

Lee [210] mentions in his work that for a system to take full advantage of a CPS’s capabilities, one must think in abstractions that allow contemplation of both the dynamic physical environment and the information processing. These abstractions must be included in platforms or models that efficiently manage the physical and software processes to achieve the system objectives. In addition, as mentioned by Bellman et al. [12], introducing a self-aware system’s learning and reasoning capabilities into the CPS development paradigm is challenging while keeping these capabilities relatively explicit and accessible for processing.

The challenges involved in a self-aware cyber-physical NoC infer the development of a new paradigm involving the construction of computational and communication abstractions. A layered distributed approach can help address different problems modularly, so the issues of each layer are treated independently, and changes made to a specific layer will not affect other layers’ behavior. The management of network resources and its related challenges, together with the requirements of the MPSoC development trend, has inspired the research of new solutions that harmoniously combine the advances and ideas in this field. Such is the case of the motivation of SDNoCs, which, as mentioned by Gomez-Rodriguez et al. [2], is related to the management problems it can solve.

##### SDNoC Architecture

Gomez-Rodriguez et al. [2] reviewed the literature and state-of-the-art for SDNoCs, clarifying their conceptualization and further explaining the initial motivation for the approach and the development path taken in recent years. Based on this work and given that the main feature of SDN is to simplify network management processes, we believe that an SDNoC architecture would allow the organization and host each of the features of an SA-CPSoC.

The term SDN dates to 1996 and arose to give the user control over network entities’ data forwarding [214]. Before SDN, the main disadvantages of network systems were the interoperability between network entities from different vendors and the difficulty of implementing network configurations. Thus, the main idea of SDN was to decouple control and data planes so that the control rests out of individual network entities on a centralized controller. Within this architecture, the controller makes the forwarding decisions by having an overview of the network, and then these decisions are passed to network entities like switches to execute them [1,9]. This approach directly impacted network performance by opening the possibility for the online configuration of network entities through software-based services and for the standardization of multivendor networks through open interfaces between control and data plane devices. The SDN concept lets the system define the forwarding policy based on programmable network services, which are aware of the application [214]. Therefore, SDN emerged as a solution to simplify network management for the new intelligent applications requiring dynamic functions with reduced operational and maintenance costs [1].

More recently, Sandoval-Arechiga et al. [80] proposed introducing the SDN concept into the NoC field and leveraging its advantages within the MPSoCs. Since this proposal, various researchers started working on this concept due to its potential to increase the capabilities of the MPSoCs. This SDNoC architecture brings characteristics like programmability and abstraction capacity to the NoC management environment, opening the possibility for online reconfiguration and enabling design reuse. SDNoC architecture improves and simplifies NoC management, resulting in more system flexibility, reduced complexity of network entities (routers), real-time guarantees, and communication network self-adaptation [50]. It converts the routers into less-complex entities, which can be programmed by following a forwarding policy dictated by a centralized software controller.

**Figure 7:**
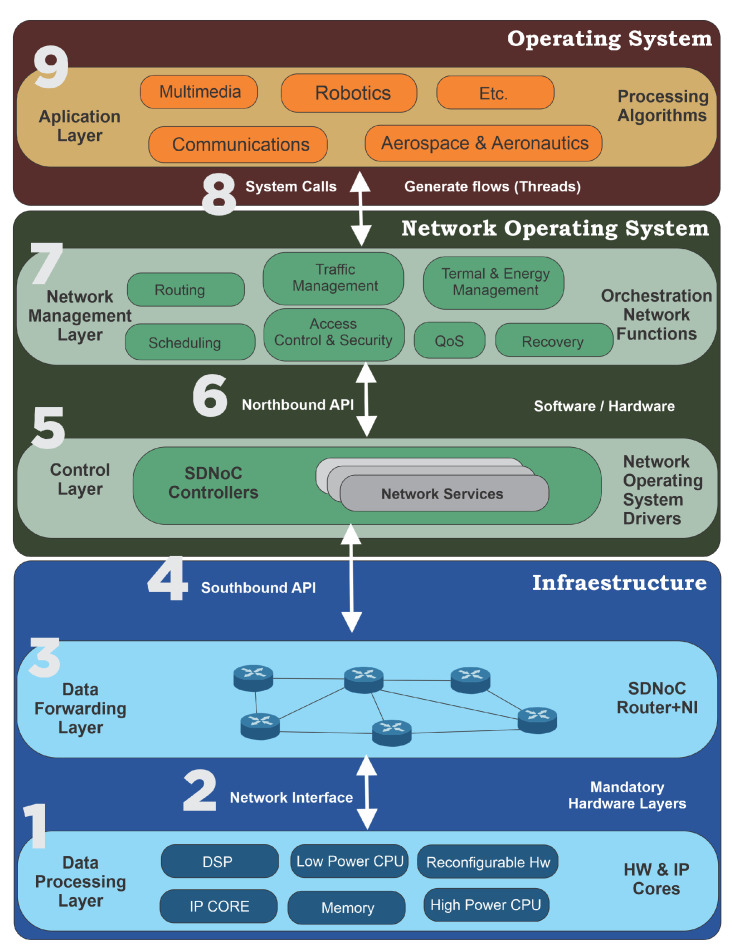
SDNoC architecture. Odd numbers represent the layers, and even numbers represent the HW/SW interfaces. A different color represents each segment [2].

The SDNoC architecture consists of three segments: application, network operating system (NOS), and infrastructure, and five layers: application, network management, control, data transmission, and data processing [2], as shown in Figure 7. This architecture has a hierarchical organization where each layer provides a service to a higher layer through a well-defined interface. In their work, Gomez-Rodriguez et al. [2] described each of this architecture’s components and their possible implementations in detail.

An SDNoC architecture provides certain facilities that can be exploited to implement the SA-CPSoC characteristics. Thus, SDNoC can be a valuable tool for constructing a self-awareness architecture for MPSoCs serving as the backbone of SA-CPSoCs. The layered infrastructure and abstraction allow a more straightforward connection with the monitoring and acting infrastructure. The network operating system (NOS) and the optimization machines help with the concentration of information and the definition of policies to specify more efficient processes or tasks depending on the system status and application. Communication channels and protocols are already created and ready to use, so a new service or element can be included just by adding an existing well-defined interface or controller. In this way, the SDNoC architecture facilitates the orchestration of physical and software processes through scalable administrative functions and SDNoC controllers.

A development challenge of this approach is the scalability problem using a centralized SDN controller, which can impact the performance of large-scale MPSoCs. Some researchers have proposed a distributed organization for the SDNoC controller to leverage the multiple advantages of distributed systems. Table 7 shows SDNoC research according to the controller organization and system’s goal. In this way, SDNoC facilitates the communication infrastructure over a distributed system, which is one of the most critical elements of such systems. Having well-defined communications protocols for a distributed system allows new services to be set up, leveraging that backbone of communication infrastructure. For example, supposing the system requires a new thermal monitor service, it can be added by connecting to the communication infrastructure straightforwardly using standardized interfaces and just focusing on the controller design for upper layers.

From this perspective, we think that an SDNoC architecture could be an effective tool or even the main baseline for the SA-CPSoCs. There are still many details to define and other development challenges this proposal brings, but the SDNoC architecture can potentially solve many of the future MPSoC problems.

## 5. Conclusions

After an extensive investigation of the state-of-the-art management within the MPSoC field over the last twenty years, this paper presents a classification of management types based on some of the issues that have driven the development of MPSoCs. The research also analyzes the optimization or improvement objectives of the research papers, identifying trends that show the importance and impact of the most exploited areas and those that are becoming increasingly relevant. Additionally, the paper identifies research papers that implement self-x properties and classifies them according to the specific type of awareness they implement to illustrate the evolution of the research and the precedents of the idea of SA-CPSoCs.

The paper describes the evolution of ideas, concepts, and developments before the conception of SA-CPSoCs as a solution to the demands of new and future MPSoCs and presents the challenges that this task implies. The paper also presents a network-based management of MPSoC that leverages the SDNoC architecture characteristics to strengthen the development of SA-CPSoCs as a conceptual idea.

The main objective of this research is to provide the scientific community with a primary point of reference in MPSoCs management and the integration of self-awareness in this field. This comprehensive and structured material will facilitate future research and developments.

## Figures and Tables

**Table 1:** Classification of management research papers of the last twenty years.

		Organization			Focus		
		Centralized	Distributed	Hierarchical	Hardware	Software	Hardware & Software
Attendable Issue	Scalability	[9,23,31,32,33,34,35,36,37,38,39,40,41]	[4,9,15,24,31,38,42,43,44,45,46,47,48,49,50,51,52,53,54,55]	[18,27,31,43,45,56,57] [2,15,32,37,38,47,51,53]	[34,39,53,58]	[24,31,42,45,47,59] [15,37,38,40,49,54]	[18,27,43,57,60] [2,33,41,48,51,55,61,62]
	Runtime Management	[25,31,63,64,65,66,67,68,69,70,71] [11,17,21,35,38,72,73,74,75,76,77,78,79,80,81,82,83,84,85,86,87,88,89,90,91,92,93,94,95,96,97,98]	[24,31,42,43,69,99,100,101,102,103,104] [26,30,46,47,84,105,106,107] [29,38,51,55,89,108,109,110,111] [14,15,53,94,112,113,114]	[31,65,69,101,115,116] [26,30,43,73,117,118] [28,38,47,51,53,93,108] [11,15,97,113,114,119]	[65,73,103,120] [88,110,121] [10,53]	[16,31,59,63,64,122,123,124,125] [24,26,42,74,75,77,78,116] [47,81,84,85,117,126,127,128,129] [15,38,91,98,108,109,112,113,114]	[25,43,66,68,69,100,101,102] [17,72,76,79,104,105,107] [28,30,55,82,83,86,87,92] [20,21,51,93,97,111,119,130]
	Architectural Improvement	[68,73,131,132,133] [17,33,80,82,83] [1,87,93,134,135] [21,40,97,98,136]	[99,100,101,131,137,138] [22,43,45,105,107,139] [52,53,55,113,140,141]	[18,101,115,132,133,137] [22,28,43,45,73,138] [53,93,97,113,119]	[58,73,142] [121,139,140] [1,53,141]	[16,125,131] [45,143] [40,98,113]	[18,19,100,101,132,133,137] [17,22,43,68,105,107,144] [28,33,55,82,83,87,134] [12,21,61,93,97,119]

**Table 2:** Classification of management research papers according to their optimization objective or improvement.

Year	Management Goal or Improvement										
	Power Efficiency	Thermal	Latency	Fault-Tolerance	Throughput	Security	QoS	Execution Time	Area	NoC Focused	Self-x Properties
2001	[152]	[152]									
2003	[122]	[122]	[153]		[153]					[153]	
2004	[60]	[60,99]			[60,131]					[99,131]	
2005	[100,154]	[64]			[63]					[63]	[100]
2006	[123]				[56]		[56]			[56]	
2007	[155,156]	[124,155]	[66]	[137]	[115]		[115]	[66]	[66]	[66,115,137,156]	[115,137]
2008	[16,58,157]	[59]	[19]	[158]						[19,58,158]	
2009	[67,159]	[67]	[160]							[160]	[67]
2010	[161,162]	[31,125,161]	[71]	[25,101]	[125,161,162]					[71,161,162]	[25,31,71,101]
2011	[70,103]	[70,103]	[116,163]	[69]	[163]		[102,116,138]			[102,116,163]	[70]
2012	[43]	[142]	[73]	[72]	[142]					[43,73,142]	[43,72]
2013	[75,164]	[165,166]	[104,164,166]	[74]	[74,164,165,166]		[164]	[24,74,76]	[165]	[76,104,164,165,166]	[74,75]
2014	[167]	[167]								[167]	[167]
2015	[79,106]	[77,79,143]	[106,168,169]	[26,120,168,169]	[78,168]		[79]	[78,170]		[77,106,143,168,169,170]	[26,78,79,120]
2016	[22,44,82,127,171]	[22,126]	[82,172]	[57]	[107]	[45,139]		[81,127]	[44]	[22,44,45,82,107,139,172]	[57,107]
2017	[32,34,46,86,118,173]	[86]	[32,33,34,118,140]	[30,34,117] [86,121,134]	[34,86,118]	[34,84]	[86]	[85]	[33,174]	[32,33,34,121,134] [140,173,174]	[30,85,117,121]
2018	[4,92,175,176,177] [89,90]	[89,92,147,176]	[4,87,176,177]	[47,48,177,178,179]	[4]	[1,88]	[87,91,92]	[108]		[1,4,87,89,176] [91,147,179]	[1,4,89,177,178] [91]
2019	[38,40,51,52,180] [9]	[146,181]	[29,49,50,180,182]	[141,183]	[180]	[37,39,110,135]	[29,109]	[40,53]	[9]	[37,50,141,146,180] [9,39,52,110,135,181] [49,182,183]	[38,50,51,109,146] [29,181]
2020	[136,184,185,186,187] [20,114,145,188]	[96,112,145,187,189] [20,148]	[10,14,55,95,190]	[14,96,113,119,149]	[10,148,188,189]	[21,94,149]	[14]		[188,191]	[21,94,95,149,191] [10,14,148,185,188,189,190]	[21,94,119,136,191] [14,113,148]
2021	[11,54,98,192]	[98,193]	[54,192,194]	[15,195]	[54]	[41,196]	[197]			[41,54,192,193,194,195,196]	[11,15,195,197]
2022	[128,198,199]	[111]	[61]	[128]		[198]		[61,128,199]	[198]	[61,111,128,198,199]	[61,111,128,198]
2023	[129,130,200]	[129,201]	[62]	[130,202]						[62,201]	[62,129,130,201,202]

**Table 3:** Analysis of research trends according to Table 2.

Management Goal or Improvement	Last 20 Years		Last 15 Years		Last 10 Years		Last 5 Years		Trend
	Number of Papers	Percentage	Number of Papers	Percentage	Number of Papers	Percentage	Number of Papers	Percentage	
Power effieciency	66	37.93%	55	36.67%	46	38.02%	25	37.88%	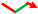
Thermal	42	24.14%	34	22.67%	25	20.66%	14	21.21%	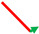
Latency	40	22.99%	37	24.67%	29	23.97%	15	22.73%	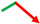
Fault-Tolerance	35	20.11%	33	22.00%	28	23.14%	12	18.18%	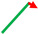
Throughput	28	16.09%	22	14.67%	13	10.74%	6	9.09%	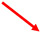
Security	16	9.20%	16	10.67%	16	13.22%	10	15.15%	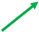
QoS	15	8.62%	13	8.67%	9	7.44%	4	6.06%	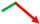
Execution time	15	8.62%	14	9.33%	11	9.09%	5	7.58%	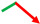
Area	9	5.17%	8	5.33%	7	5.79%	4	6.06%	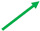
NoC Focused	97	55.75%	85	56.67%	70	57.85%	40	60.61%	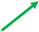
Self-x properties	58	33.33%	55	36.67%	45	37.19%	28	42.42%	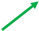

Note: The percentage of each metric considers the total number of related papers within the specified time range. There are research papers related to more than one metric.

**Table 4:** Classification of the NoC-related papers according to their main optimization metric and the most common specific NoC management improvement area.

	Management Goal or Improvement								
NoC Management Improvement Area	Power Efficiency	Thermal	Latency	Fault-Tolerance	Throughput	Security	QoS	Execution Time	Area
Routing algorithm	[161,180,188]	[165,189,193] [161,166]	[10,50,62] [49,168,180] [104,165,166] [71,153,160] [169,182,194]	[134,168,179] [169,195]	[10,188,189] [107,168,180] [115,161,166] [153]	[21,37,41]	[115]		[165,188]
Topology	[4,44,199] [22,172,173,192]	[22,77]	[4,116,160] [169,182,190,192]	[169,183,203]	[4,107]		[116]	[170,199]	[44]
Buffer	[52,198]		[10]		[10,115]	[198]	[115]		[174,198]

**Table 5:** Classification of research papers according to their specific awareness.

Year	Aware System Management												
	Thermal-Aware	Energy-Aware	Reliability-Aware	Traffic-Aware	Congestion-Aware	Environment-Aware	Application-Aware	Workload-Aware	Contention-Aware	QoS-Aware	Loss-Aware	Fluidity-Aware	NoC Focused
2003					[153]								[153]
2004				[131]	[131]	[132]							[131]
2005		[64,154]		[63]				[100]					[63]
2006						[133]							
2007	[124]	[156]			[66]								[66,156]
2008		[58]					[158]						[58]
2010	[31,125,161]			[71,161]	[71]	[31]							[71,161]
2011	[70,103]					[68]	[68]		[163]	[138]			[163]
2012					[142]								[142]
2013	[104,165,166]			[76,104,165]			[164]						[76,104,164]
2015	[79,143]		[168]	[77]			[168]	[78]	[106]				[77,106,168]
2016	[126]			[82]	[82]			[81]					[82]
2017			[86,117]					[30]					
2018	[147,176]		[179]			[28]	[1,28]	[179]	[176]				[1,28,147,179]
2019	[146,181]	[52]			[180]		[29]	[38]	[40]	[29,109]			[52,146,181]
2020	[96,112,189]	[97,184,186]	[96,184,191]	[10,95,189]	[10,189]	[119,187,207]		[119]			[185]	[10]	[10,95,185,189,191]
2021	[98,148]	[98]	[197]						[193]				[148,193]
2022	[111]	[111,198,199,208]	[111,128]		[199]								[111,128,198,199]
2023	[129]		[130,201,202]		[62]								[62,201]

**Table 6:** Challenges facing the development of SA-CPSoCs [12,206].

Challenges			
Self-Awareness [206]	What Is Needed?	SA-CPSoC [12]	What Is Needed?
Dynamic Learning	Better machine learning algorithms based on feedback signals.	Considering self-awareness, subjectivity, and situatedness.	Techniques that consider the system’s own perspective in different possible situations in addition to the environmental changes. Enhancing the decision-making process.
Scalable self-awareness	Define different levels of self-awareness for different system requirements.	Building resource-sensitive self-awareness.	Consider the resources needed to implement self-awareness and its processes at runtime.
Ensuring correctness	Validate the level of systems adaptation ensuring reliability and guarantees.	Verifying self-awareness and establishing guarantees.	Methods to implement verifications of self-awareness level from the design stage and make the system understand the guarantees during operation.
Design methology	Change the design paradigm to let the systems be self-aware.	Developing new designs and engineering processes.	Adapt design and processes to introduce self-aware CPSs characteristics including dynamic decisions instead of predefined decisions.
Formulation goals	Define more quantitative goals like adaptability, autonomy, self-assessment, and situation assessment, and formulating mechanisms to define trade-offs.	Creating an infrastructure for self-awareness processes.	New reference architectures and design templates guided to provide a generic infrastructure that facilitates the development of SA-CPS and all of its capabilities.

**Table 7:** SDNoC research according to the controller organization and system’s goal.

		Management Goal or Improvement						
		General	Power Efficiency	Latency	Fault-Tolerance	Throughput	Security	QoS
Organization	Centralized	[23,35,36] [80,83]	[9,32]	[32,33,87]	[149]		[1,21,37] [41,94,149]	[87,91]
	Distributed		[4,31,44]	[14,29,50] [4]	[14]	[4]	[1,49,94]	[14,29]

## Data Availability

The original contributions presented in the study are included in the article, further inquiries can be directed to the corresponding author.
